# Regulation of chicken vanin1 gene expression by peroxisome proliferators activated receptor α and miRNA-181a-5p

**DOI:** 10.5713/ajas.19.1000

**Published:** 2020-04-12

**Authors:** Zhongliang Wang, Jianfeng Yu, Nan Hua, Jie Li, Lu Xu, Wen Yao, Zhiliang Gu

**Affiliations:** 1 School of Biology and Food Engineering, Changshu Institute of Technology, Changshu, 215500, Jiangsu, China; 2 College of Animal Science and Technology, Nanjing Agricultural University, Nanjing, 210095, Jiangsu, China

**Keywords:** Chicken, Vanin1 Gene, Peroxisome Proliferators Activated Receptor α(PPARα), miRNA-181a-5p, Regulatory Mechanism

## Abstract

**Objective:**

Vanin1 (VNN1) is a pantetheinase that can catalyze the hydrolysis of pantetheine to produce pantothenic acid and cysteamine. Our previous studies showed that *VNN1* is specifically expressed in chicken liver. In this study, we aimed to investigate the roles of peroxisome proliferators activated receptor α (PPARα) and miRNA-181a-5p in regulating *VNN1* gene expression in chicken liver.

**Methods:**

5′-RACE was performed to identify the transcription start site of chicken *VNN1*. JASPAR and TFSEARCH were used to analyze the potential transcription factor binding sites in the promoter region of chicken *VNN1* and miRanda was used to search miRNA binding sites in 3′ untranslated region (3′UTR) of chicken *VNN1*. We used a knock-down strategy to manipulate PPARα (or miRNA-181a-5p) expression levels *in vitro* to further investigate its effect on *VNN1* gene transcription. Luciferase reporter assays were used to explore the specific regions of VNN1 targeted by PPARα and miRNA-181a-5p.

**Results:**

Sequence analysis of the VNN1 promoter region revealed several transcription factor-binding sites, including hepatocyte nuclear factor 1α (HNF1α), PPARα, and CCAAT/enhancer binding protein α. GW7647 (a specific agonist of PPARα) increased the expression level of *VNN1* mRNA in chicken primary hepatocytes, whereas knockdown of PPARα with siRNA increased VNN1 mRNA expression. Moreover, the predicted PPARα-binding site was confirmed to be necessary for PPARα regulation of *VNN1* gene expression. In addition, the *VNN1* 3′UTR contains a sequence that is completely complementary to nucleotides 1 to 7 of miRNA-181a-5p. Overexpression of miR-181a-5p significantly decreased the expression level of *VNN1* mRNA.

**Conclusion:**

This study demonstrates that PPARα is an important transcriptional activator of *VNN1* gene expression and that miRNA-181a-5p acts as a negative regulator of *VNN1* expression in chicken hepatocytes.

## INTRODUCTION

Vanin-1 (*VNN1*) is a type of pantetheinase that is primarily expressed in the mammalian liver, kidney, heart and gut. *VNN1* can catalyze the hydrolysis of pantetheine to produce pantothenic acid (vitamin B_5_) and cysteamine (a highly effective antioxidant) [1]. Among the three orthologous Vanin genes (i.e., *VNN1*, *VNN2*, and *VNN3*), *VNN1* is the most prevalent and important isoform and is involved in inflammation, oxidative stress and cell migration [2–4]. Recently, it has been revealed that *VNN1* is closely related to fatty acid metabolism. Van Diepen et al [5] found that RNAi-induced *VNN1* knockdown in mice aggravated the accumulation of liver triglycerides (TGs). Similarly, rats treated with the VNN1 inhibitor RR6 exhibited more severe liver TG accumulation in response to fasting [5]. Our previous studies showed that the chicken *VNN1* gene is specifically expressed in the liver [6]. Functionally, whether the clustered regularly interspaced short palindromic repeats (CRISPR)-CRISPR–associated protein 9 (Cas9) is used to mediate knockout of the *VNN1* gene in chicken Leghorn male hepatoma (LMH) cells or RNAi is used to mediate knockdown of the *VNN1* gene in primary chicken hepatocytes, transcriptome sequencing results show that lipid metabolism-related pathways are widely enriched and that lipid metabolism-related genes (such as apolipoprotein A4, ELOVL fatty acid elongase 2, and fatty acid synthase) are differentially expressed (unpublished data). These findings indicate that *VNN1* is a potential factor involved in the regulation of fatty acid metabolism and lipid homeostasis.

In mammals, the expression of *VNN1* is affected by external factors such as fasting, drugs, circadian rhythm, and oxidative stress [5,7,8] and is also regulated by some internal regulatory factors. Most recently, Chen et al [7] revealed that hepatocyte nuclear factor 4α [HNF4α] promotes the transcription of the *VNN1* gene by binding to two HNF4α-binding sites in the mouse *VNN1* gene promoter. Our studies have shown that the *VNN1* gene is significantly upregulated by miR-122 knockdown in chicken [9]. However, studies on the transcriptional and posttranscriptional regulation mechanisms of the chicken *VNN1* gene have not yet provided clear results. Therefore, it is necessary to conduct an in-depth study of the regulation of *VNN1* expression, providing new insights into the mechanism of regulation within chicken lipid metabolism.

Peroxisome proliferators activated receptor α (PPARα), a member of the nuclear hormone receptor superfamily that is ubiquitously expressed in various tissues but is mainly enriched in tissues with high fatty acid oxidation rates, such as the rodent liver [10]. PPARα is currently considered a nutrient sensor and has been identified as the master regulator of hepatic lipid metabolism in response to feeding and starvation [11]. Several different research groups have indicated that PPARα can induce a robust increase in *VNN1* expression in the mouse liver and strongly modulate plasma Vanin activity. Moreover, liver-specific knockdown of *VNN1* improves hepatic steatosis in *db/db* and diet-induced obese mice [7,12]. However, these findings are descriptive, and the detailed mechanism by which PPARα regulates the chicken *VNN1* gene remains unknown.

MicroRNAs (miRNAs), are a class of endogenous noncoding small regulatory RNAs of approximately 18 to 25 nucleotides in length that modulate the expression of their target genes to produce a posttranscriptional regulation pattern in animals [13,14]. Currently, it has been widely reported that miRNAs facilitate the degradation of target genes through complementary binding between the seed regions (ranging from the 2nd to 8th nts) of miRNAs and the 3′-untranslated regions (UTRs) of their target genes [15]. Related reports have revealed that more than 60% of all human genes could be directly or indirectly regulated by miRNAs [16]. Recent advances have shown that miRNAs play crucial roles in different biological processes (such as apoptosis, cell proliferation, organismal development) and many diseases, including cancer and obesity-related diseases [17,18]. In addition to different biological processes and diseases, miRNAs are also linked to different types of biological metabolism, such as glucose metabolism and lipid metabolism [19,20]. In chickens, bioinformatics analysis showed that the *VNN1* gene is a target gene of miR-122, and subsequent dual luciferase experiments revealed that miR-122 can bind to a partial sequence in the 3′UTR of *VNN1* and act as a negative regulator [6]. Therefore, the possibility that other miRNAs serve as endogenous posttranscriptional regulatory factors to affect the expression of *VNN1* cannot be reasonably excluded. Based on the findings mentioned above, we aimed to investigate the role of PPARα and miRNA-181a-5p in the regulation of the chicken *VNN1* gene. To achieve this goal, we used a loss-of-function strategy to manipulate PPARα (or miRNA-181a-5p) expression levels *in vitro* based on the delivery of a PPARα agonist (or miRNA-181a-5p-mimics) and small interfering RNA (siRNA) to further investigate its effect on *VNN1* gene expression. Mechanistically, luciferase reporter assays and site-directed mutagenesis techniques were used to explore the specific mechanisms by which PPARα (or miRNA-181a-5p) regulates the *VNN1* gene. In this paper, we provide experimental evidence suggesting that PPARα plays an important role in the transcriptional activation of the *VNN1* gene and that miRNA-181a-5p acts as a negative regulator to regulate *VNN1* gene expression in chicken hepatocytes.

## MATERIALS AND METHODS

### Animal care and experimental procedures

The use of animals, the procedures for animal management, and the collection of animal tissues in this experiment were approved by the Animal Care Committee of the Changshu Institute of Technology (Permit number: EAWEC1710). Commercial broiler chickens were housed under the conditions of ~25°C, ~60% humidity, and 16 h light-8 h darkness with free access to water and feed. For the fasting group, chickens were fasted for 24 h, and for the refeeding group, the chickens were refed for 2 h after 24 h of fasting. Liver samples were immediately dissected, snap-frozen in liquid nitrogen, and stored at −80°C until further processing.

### Rapid amplification of 5′-cDNA ends (5′-RACE)

5′-RACE was performed using the 5′-Full RACE Kit (TaKaRa, Dalian, China) to identify the transcription start site (TSS) of chicken *VNN1*. Briefly, total RNA was extracted from chicken hepatocytes using TRIzol (Invitrogen, Carlsbad, CA, USA) following the manufacturer’s instructions and then treated with calf intestine alkaline phosphatase (CIAP) and tobacco acid pyrophosphatase (TAP). The CIAP/TAP-treated RNA and the 5′ RACE adaptor were ligated with T4 ligase. 5′RACE cDNA was synthesized from the adapter-ligated RNAs using M-MLV reverse transcriptase and random 9 mers. The first round of polymerase chain reaction (PCR) amplification was carried out using the 5′ RACE cDNA as a template and the 5′ RACE Outer Primer/gga-*VNN1*-spR1 primers, followed by the second round of PCR with the 5′ RACE Inner Primer and gga-*VNN1*-spR2. The PCR products were separated in 1.5% agarose gels and then cloned into the pMD19T vector for sequencing.

### Sequence analysis

The AA sequences of *VNN1* from chicken (NP_001034377.1_Gallus_gallus), humans (CAA10568.1_Homo_sapiens), mouse (NP_035834.2_Mus_musculus), rabbit (XP_002714789.1_Oryctolagus_cuniculus), sheep (XP_014952903.1_Ovis_aries), goose (ASA40330.1_Anser_anser_domesticus), swan goose (XP_013030943.1_Anser_cygnoides_domesticus), duck (XP_005009624.2_Anas_platyrhynchos), pig (NP_999298.1_Sus_scrofa), cattle (NP_ 001019727.2_Bos_taurus), toad (XP_004914646.1_Xenopus_tropicalis), bear (XP_026360 602.1_Ursus_arctos_horribilis), monkey(XP_017735082.1_Rhinopithecus_bieti), killer whale (XP_004263864.1_Orcinus _orca), and giant panda (XP_011 227948.1_Ailuropoda_melanoleuca) were obtained from the NCBI database. Multiple alignment of sequence homology was performed using DNAMAN9.0. The phylogenetic tree was constructed using MEGA7. The TSS of chicken *VNN1* was determined based on the results of 5′-RACE. The promoter region of the chicken VNN1 gene (1,000 bp upstream of the TSS) and the 200 bp downstream sequence of the TSS were obtained from the NCBI database. The 1,200 bp 5′-regulatory region was analyzed for potential transcription factor-binding sites using JASPAR (http://jaspar.genereg.net/) and TFSEARCH (http://www.cbrc.jp/research/db/TFSEARCH.html). The 3′UTR sequence of chicken *VNN1* was downloaded from the 3′UTR database (http://utrdb.ba.itb.cnr.it/). The miRNA target prediction software miRanda was employed to predict miRNA-binding sites in the chicken *VNN1* 3′UTR.

### Construction of *VNN1* promoter-luciferase/3′UTR-luciferase constructs

To detect *VNN1* promoter activity, the chicken *VNN1* gene fragment (−1,000/+127) was amplified by PCR from the chicken liver genome using the primers gga-*VNN1*-F1 and gga-*VNN1*-R. The TSS position was +1, and the downstream primer 3′ end was located at +127. After digestion and sequencing, the DNA fragment was subcloned into the luciferase reporter vector pGL3 promoter (the construct was named pGL3-p-1127). The resulting pGL3-p-1127 plasmid was used as a template to further amplify a series of 5′-deleted fragments using the following primers (gga-*VNN1*-F2, gga-*VNN1*-F3, gga-*VNN1*-F4, and gga-*VNN1*-R). All primers for the five 5′-deletion constructs included a cleavage recognition site (indicated in bold font), which was a *Kpn* I site in the forward primers and a *Hind* III site in the common reverse primers, to facilitate subcloning into the *Kpn*I/*Hind*III site of the pGL3-basic vector (Promega, Madison, WI, USA). After identification by enzyme digestion and sequencing, five 5′-deletion promoter reporter plasmids were successfully constructed. According to the length of the amplified fragments, they were named pGL3-p-1127, pGL3-p-716, pGL3-p-548, pGL3-p-263, and pGL3-p-143. A PPARα-binding site mutation (PPARα-Mut) was generated from construct pGL3-p-263 by overlap-extension PCR using the following mutagenesis primers (PPARα-Mut-Forward and PPARα-Mut-Reverse, the mutated bases are underlined). An expression plasmid encoding chicken PPARα (pcDNA3.1 (+) - PPARα) was constructed in the early stage of the experiment.

To further study the posttranscriptional regulation mechanism of the *VNN1* gene, a luciferase reporter gene vector containing the *VNN1* 3′ UTR was constructed. In brief, the fragment of the chicken *VNN1* 3′UTR encompassing the predicted miR-181a-5p-binding site was amplified by PCR and directionally inserted downstream of the luciferase expression cassette of the pMIR-reporter vector (Ambion, Carlsbad, CA, USA) to construct the pMIR-*VNN1*-3′UTR reporter vector. The pMIR-*VNN1*-3′UTR-mut reporter plasmid was generated using overlap-extension PCR. In this construct, the seed region of the predicted miR-181a-5p-binding sequence within the chicken *VNN1* 3′UTR was mutated. All recombinant vectors were extracted with PureLink HiPure Plasmid Filter Purification Kits (Invitrogen, USA) and verified by sequencing. The primer sequences used for the cloning of the promoter or 3′ UTR and plasmid construction are shown in Supplementary Table S1.

### RNA extraction and real-time polymerase chain reaction

Total RNA was isolated from LMH cells, primary chicken hepatocytes and liver tissue using RNAiso Plus (TaKaRa, China) according to the manufacturer’s protocol and treated with RNase-free DNase. The concentrations of RNA were determined using a NanoDrop ND2000 spectrophotometer (Thermo Scientific, Wilmington, DE, USA). For reverse transcription, 0.5 μg of extracted RNA per sample was reverse-transcribed using the PrimeScript RT reagent kit (TaKaRa, China) following the manufacturer’s instructions. cDNA was amplified using SYBR Green PCR Master Mix (Applied Biosystems, Foster City, CA, USA) in an ABI Prism 7500 sequence detection system (Applied Biosystems, USA). The primers used for real-time PCR are listed in Supplementary Table S1. Quantitative PCR (qPCR) was performed in triplicate for each cDNA sample, and the results were normalized to endogenous actin mRNA. The expression of miR-181a-5p was quantified by RT-qPCR according to the protocol of the TaqMan MicroRNA Assay (Applied Biosystems, USA) and was normalized to chicken 18S rRNA. All the reactions were performed in duplicate. The data were analyzed using the 2^−ΔCT^ method or the 2^−ΔΔCT^ method.

### Cells, cell isolation and culture

The chicken LMH cell line (ATCC) has well-differentiated morphological and biochemical characteristics and has been widely used as a good cell model for studying chicken liver lipid metabolism. LMH cells were maintained in Waymouth’s medium (Gibco, New York, NY, USA) containing 10% fetal bovine serum (Gibco, USA) and 100 U/mL penicillin/streptomycin in a humidified incubator (37°C, 5% CO_2_). Chinese hamster ovary (CHO) cells were maintained in Ham’s F-12K medium (Gibco, USA) containing 10% fetal bovine serum (Gibco, USA) and 100 U/mL penicillin/streptomycin in a humidified incubator (37°C, 5% CO_2_). Chicken hepatocytes were isolated using a modified two-step collagenase method as previously described. The isolated hepatocytes were seeded at a density of 8×10^5^ cells/mL in William’s E medium (Gibco, USA) supplemented with 5% chicken serum, 100 U/mL penicillin-streptomycin, 10 μg/mL insulin and 30 mM NaCl.

### Plasmid transient transfections and luciferase assay

To detect *VNN1* promoter activity, LMH cells (1.5×10^5^ cells/well) were plated in 24-well plates for 24 h and grown to ~70% confluence in antibiotic-free medium before transfection. The cells were transfected with 250 ng/well of the 5′-deletion promoter reporter plasmids, the pGL3-basic empty vector or the pGL3-promoter strong promoter vector using X-tremeGENE 9 at a transfection reagent/DNA ratio of 3:1. pRL-CMV (Renilla luciferase) was used as an internal control to normalize the transfection efficiency. Forty-eight hours after transfection, the cells were harvested and analyzed for luciferase activity using a Dual-Luciferase Reporter Assay System (Promega, USA) according to the manufacturer’s protocol. To further verify the effect of the transcription factor PPARα on *VNN1* promoter activity, pcDNA3.1(+)-PPARα and pGL3-p-263 or pGL3-p-263-mut were cotransfected into LMH cells cultured in 24-well plates. All experiments were performed in triplicate wells and repeated in three independent trials. Luciferase activity was normalized to the Renilla luciferase activity. To determine the targeting relationship between miRNA-181a-5p and the *VNN1* 3′UTR, CHO cells were seeded in 24-well plates for 24 h before transfection. pMIR-*VNN1*-3′UTR or pMIR-*VNN1*-3′UTR-mut (200 ng), miRNA-181a-5p-mimic or negative control (NC) mimic (20 μM) and the internal control pRL-CMV (2 ng) (Renilla luciferase) were mixed and cotransfected into the cells using the Lipofectamine 2000 reagent (Invitrogen, USA). After transfection for 48 h, the cells were harvested; luciferase activities were measured using the Dual-Luciferase Reporter Assay System (Promega, USA); and the firefly luciferase signal was normalized to the Renilla luciferase signal.

### SiRNAs and miRNA-181a-5p-mimic transfection

The isolated hepatocytes or LMH cells were grown in 12-well plates until they reached confluency of 80% and were then transiently transfected with the PPARα-siRNAs (or miRNA-181a-5p-mimic) and NC-mimic according to the manufacturer’s instructions for RNAiMAX (Invitrogen, USA). After 48 h of transfection, total RNA was extracted and analyzed by RT-qPCR.

### Statistical analysis

All data are presented as the mean±standard error of the mean of at least three independent experiments. Statistical analyses were performed using Student’s t-test or one-way analysis of variance. A p-value <0.05 was considered statistically significant unless otherwise noted.

## RESULTS

### Chicken *VNN1* sequence and phylogenetic tree analysis

**Figure 1:**
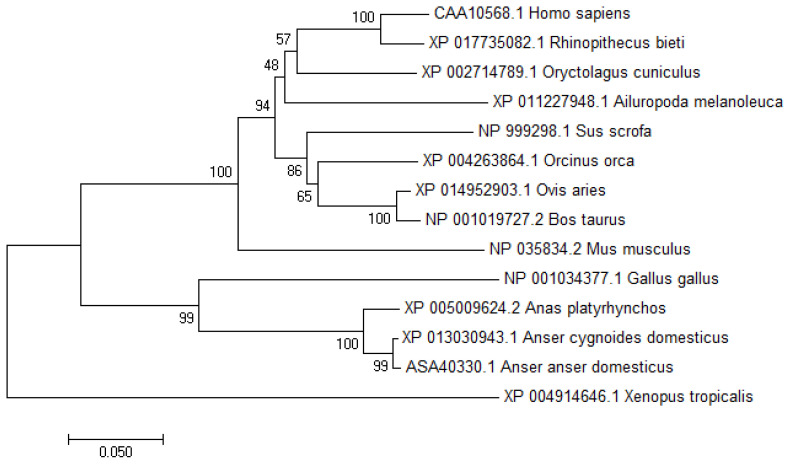
Phylogenetic tree analysis of vanin1 (*VNN1*) among multiple species. The VNN1 protein sequences of multiple species were obtained from the NCBI database. The tree was constructed with the neighbor-joining method using MEGA7.0 with 1,000 bootstrap replicates. The bootstrap confidence values were marked at the nodes.

To study the transcriptional regulation mechanism of the promoter region of the chicken *VNN1* gene, this experiment used evolutionarily conserved region (ECR) browser to analyze the conservation of the *VNN1* gene and its upstream sequence among multiple species. The results showed that the exons and ECRs of the chicken *VNN1* gene were highly conserved in relation to the human, mouse, rat and rhesus monkey sequences. The upstream sequence of the chicken *VNN1* gene did not show a conserved region shared with other species. Compared with the AA sequence (ID:NP_0010 34377.1) of chicken *VNN1*, the AA sequences of duck, goose, human, mouse, cattle, sheep, pig and toad shared 73.9%, 77.3%, 61.8%, 63.4%, 63.8%, 63.8%, 61.6%, and 60.4% sequence similarity respectively. In the phylogenetic tree, analysis of the potential evolutionary history of the *VNN1* protein among multiple species showed that chicken *VNN1* was most closely related to that of goose and presented the lowest relatedness to that of toad among all the species evaluated in this study (Figure 1).

### Characterization of chicken *VNN1* gene transcription start site and 5′-regulatory region

**Figure 2:**
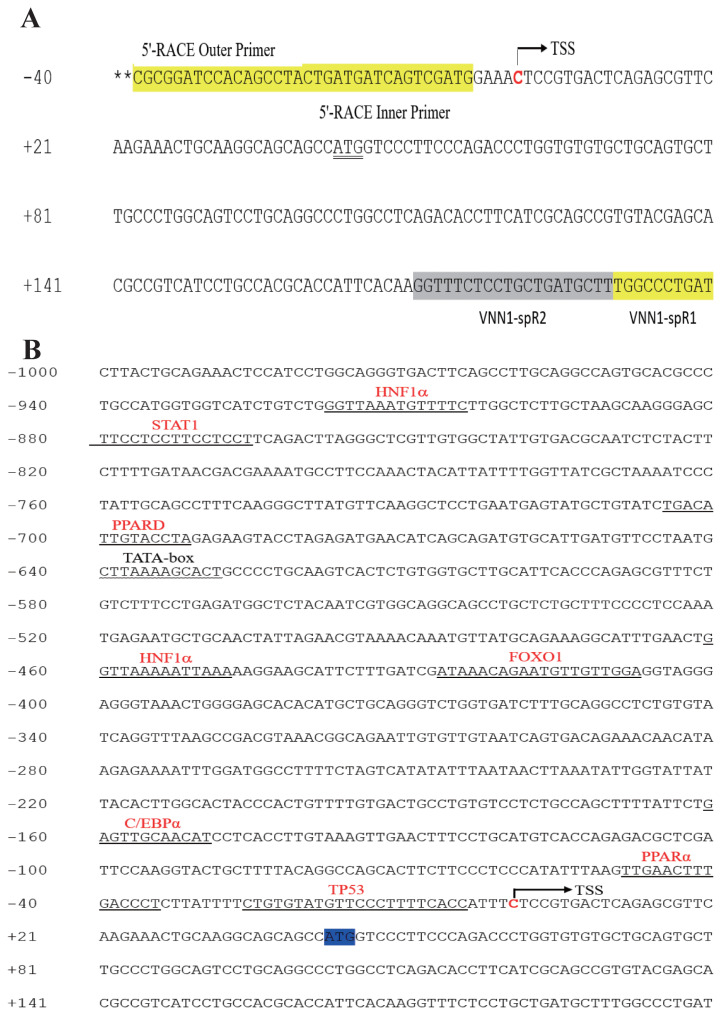
Characterization of chicken vanin1 (*VNN1*) gene transcription start site (TSS) and 5′-regulatory region. (A) Schematic representation of the primers used for the nested PCR and their relative positions complementary to sequences of the *VNN1* gene labeled in yellow. The TSS (the red base “C”) of the *VNN1* gene is located 42 bp upstream of the translation initiation codon ATG. (B) Numbers are relative to the TSS (+1). The putative regulatory elements are indicated by red letters above the underlined sequence.

To determine the TSS of the *VNN1* gene, 5′-RACE was used as described in the “Materials and methods”. Compared with the chicken *VNN1* genome sequence published in NCBI, the 5′UTR of the chicken *VNN1* gene is 42 bp in length, indicating that the TSS (chr3:56080813) of the *VNN1* gene is located 42 bp upstream of the translation initiation codon ATG (chr3: 56080855) (Figure 2A). To predict putative transcription factor-binding sites, prediction tools (JASPAR and TFSEARCH) were used to analyze the 1,200 bp 5′-regulatory region (1,000 bp upstream of the TSS and 200 bp downstream of the TSS). Several transcription factor-binding sites were recognized via sequence analysis of the 5′-regulatory region, including sites for hepatocyte nuclear factor 1α (−461/−448), PPARα (−49/−35) and the CCAAT/enhancer binding protein α (CEBPα) (−159/−149) (Figure 2B). Interestingly, the predicted TATA-box is located far from a TSS and is not a typical TATA-box in the eukaryotic promoter region.

### Transcriptional activity of the chicken *VNN1* gene promoter

**Figure 3:**
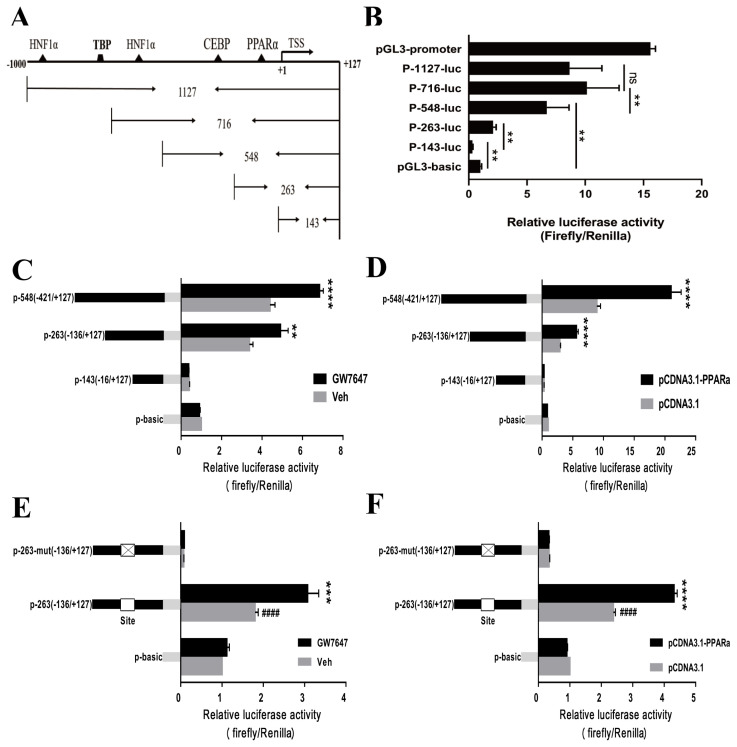
PPARα upregulates vanin1 (*VNN1*) promoter activity. (A) The truncated region corresponds to 5 different fragments of the −1,000 to +127 bp region of the *VNN1* 5′ promoter region. The locations of the HNF1α, C/EBP and PPARα sites are indicated with triangles. (B) Relative luciferase activities are represented by horizontal column length. A series of 5′-deletion constructs of the *VNN1* promoter (P-143-luc, P-263-luc, P-548-luc, P-716-luc, and P-1127-luc) fused in frame to the luciferase gene were transfected into LMH cells. Luciferase activities were determined 48 h post-transfection as described in the MATERIALS AND METHODS. Luciferase activity was normalized to Renilla luciferase activity. The experiment was performed in triplicate wells and repeated in three independent trials. The data are presented as the means±standard deviation. *, p<0.05, **, p<0.01. (C) LMH cells were transfected with the progressive 5′-deletion constructs (P-143-luc, P-263-luc, P-548-luc) for 24 h and then treated with 1 μM GW7647 or Veh for 24 h. The cell lysates were subjected to the luciferase activity assay. The data represent relative *VNN1* promoter activity normalized to pRL-CMV activity. The experiment was performed in triplicate wells and repeated in three independent trials, and the data are presented as the means± SEMs. ** p<0.01 and **** p<0.0001 vs Veh. (D) LMH cells were transfected with the progressive 5′-deletion constructs (P-143-luc, P-263-luc, P-548-luc) for 24 h along with the PPARα expression plasmid (pcDNA3.1-PPARα) or pcDNA3.1. A luciferase activity assay was performed as described above. **** p<0.0001 vs pcDNA3.1. (E) LMH cells were transiently transfected with the P-263-luc and P-263-mut-luc constructs alone for 24 h and then treated with 1 μM GW7647 or Veh for 24 h. A luciferase activity assay was performed as described above. *** p<0.001 vs the P-263-mut-luc group, #### p<0.0001 vs the P-263-mut-luc group. (F) LMH cells were transiently transfected with the P-263-luc and P-263-mut-luc constructs alone along with the PPARα expression plasmid (pcDNA3.1-PPARα) or pcDNA3.1. A luciferase activity assay was performed as described above. **** p<0.0001 vs the P-263-mut-luc group, #### p<0.0001 vs the P-263-mut-luc group. HNF1α, hepatocyte nuclear factor 1α; C/EBP, CCAAT/enhancer-binding protein; PPARα, peroxisome proliferators activated receptor α; LMH, Leghorn male hepatoma; SEM, standard error of the mean.

To identify the transcriptional activity of the chicken *VNN1* 5′-regulatory region, we constructed progressive 5′-deletion constructs of the *VNN1* promoter fused to a luciferase reporter gene (Figure 3A). LMH cells were transiently transfected with the 5′-deletion constructs of the *VNN1* promoter (P-143-luc, P-263-luc, P-548-luc, P-716-luc, and P-1127-luc). The results of the dual-luciferase reporter assay indicated that the P-143-luc construct showed less basal promoter activity than the basic-luc construct, which may have been due to the presence of negative regulatory elements in the fragment. Importantly, the relative luciferase activities of the P-263-luc and P-548-luc constructs showed very significant increases compared to that of the basic-luc construct (Figure 3B), suggesting that these fragments are essential to the promoter activity of the *VNN1* gene. The relative luciferase activity of the P-1127-luc construct was reduced compared to P-716-luc, but the difference was not significant. Additionally, compared to the P-548-luc construct, the relative luciferase activity of the P-716-luc construct was significantly increased, suggesting that a positive element may be present between p-716 and p-548 bp. These results indicated that the P-548 fragment might be a core functional promoter, which is consistent with previous findings showing that the core promoter is generally located near the transcription start site.

### PPARα plays an important role in the activation of chicken *VNN1* transcription

Since the *VNN1* promoter-263 and -548 regions are critical to promoter activity and there is a putative PPARα-binding site in these regions (Figure 3A), we next investigated whether the putative site was functional. To validate the roles of PPARα in the regulation of the *VNN1* gene, we examined the ability of GW7647 to activate the expression of each construct after transient transfection of LMH cells with the P-263-luc and P-548-luc constructs. The luciferase activity assay indicated that the *VNN1* promoter-263 and −548 regions exhibited significant increases compared with the Veh groups in response to GW7647 treatment (Figure 3C). Similar results were observed using an exogenous PPARα overexpression plasmid (Figure 3D). To further validate these results, we used chicken embryonic fibroblast cell line DF-1 as a model cell line to repeat this experiment because LMH cells express endogenous PPARα at high levels. Similar results were observed in DF-1 cells (Supplementary Figure S1). These results clearly suggest that PPARα upregulates *VNN1* promoter activity.

To further assess whether the putative PPARα-binding site is required for *VNN1* expression, we transfected wild-type (P-263-luc) and mutated promoter luciferase constructs (P-263-mut-luc) into LMH cells. As shown in Figure 3E, minimal luciferase activity was detected when the putative PPARα-binding site was mutated, regardless of the presence or absence of the PPARα agonist GW7647. LMH cells were transiently cotransfected with P-263-luc or P-263-mut-luc and the PPARα overexpression plasmid, and relative luciferase activity was measured. However, the experimental results showed that PPARα overexpression did not increase *VNN1* promoter activity (Figure 3F). These results strongly demonstrated that the predicted PPARα-binding site is indeed functional and plays a crucial role in the regulation of the *VNN1* gene.

### PPARα regulates *VNN1* gene expression

**Figure 4:**
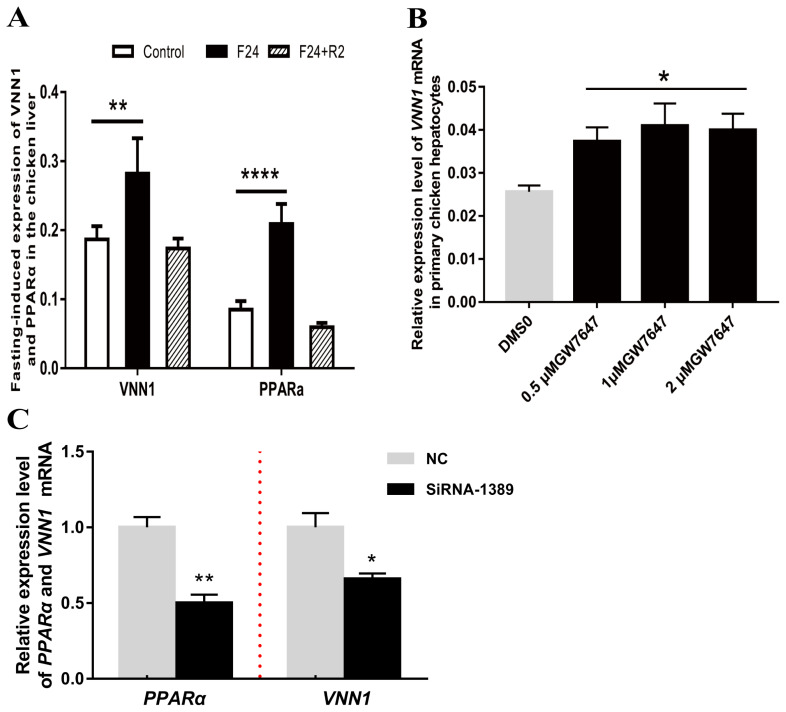
PPARα plays an important role in chicken vanin1 (*VNN1*) gene expression. (A) RT-qPCR analysis of *VNN1* and *PPARα* gene expression in the livers from chickens subjected to either fasting (24 h) alone or fasting for 24 h followed by 2 h of refeeding. *β-Actin* was used as a reference for normalization. Statistical significance is indicated as follows: ** p<0.01, **** p<0.0001, n = 8. Control, fed *ad libitum* for 24 h; F24, fasted for 24 h after 24 h of *ad libitum* feeding; F24+R2, refed for 2 h after 24 h of *ad libitum* feeding and 24 h of fasting. (B) The isolated chicken hepatocytes were treated with different concentrations of GW7647 (0.5 μM, 1 μM, 2 μM) for 24 h. Total RNA was isolated and subjected to quantitative RT-qPCR. The bars represent the means±SEMs from three independent experiments. * p<0.05 vs the DMSO group (first bar). β-Actin was used as a reference for normalization. (C) LMH cells were transfected with si-PPARα (siRNA-1389) and si-NC for 48 h, and total RNA was then isolated. The changes in the mRNA expression of *PPARα* and *VNN1* were normalized to β-actin as an internal control. The bars represent the means±SEMs from three independent experiments. * p<0.05 and ** p<0.01 vs the NC group. RT-qPCR, reverse transcription-quantitative polymerase chain reaction; *PPARα*, peroxisome proliferators activated receptor α; SEM, standard error of the mean; LMH, Leghorn male hepatoma; NC, negative controls.

PPARα is considered a crucial transcription factor that mediates the expression of numerous lipid metabolism-related genes. As shown in Figure 4A, the expression of PPARα in chicken liver was significantly upregulated after fasting for 24 h. Notably, the expression level of the *VNN1* gene also showed a very significant increase. This correlation between PPARα and *VNN1* gene expression was verified in serum-starved LMH cells (Supplementary Figure S2). We hypothesized that PPARα, a node of the lipid metabolism network, may promote the expression of the *VNN1* gene in chicken liver. To confirm this hypothesis, we treated chicken primary hepatocytes and LMH cells with GW7647, a specific agonist of PPARα. To verify whether GW7647 functions in primary chicken liver cells, RT-qPCR was used to detect the expression of the peroxisomal acyl-coenzyme A oxidase 1 (*ACOX1*) gene, a validated PPARα target gene. Our results indicated that GW7647 increased the ACOX1 mRNA expression level in a concentration-dependent manner in chicken primary hepatocytes (Supplementary Figure S3). Furthermore, we tested whether GW7647 promotes the expression of the *VNN1* gene in chicken liver cells. As illustrated in Figure 4B, GW7647 also increased the *VNN1* mRNA expression level in chicken primary hepatocytes. To further define the role of PPARα in the expression of *VNN1*, PPARα was knocked down with siRNA in LMH cells. Forty-eight hours after siRNA transfection, total RNA was isolated, and the expression levels of PPARα and *VNN1* mRNA were measured using real-time quantitative PCR. Knockdown of PPARα with siRNA significantly decreased *VNN1* mRNA expression levels (Figure 4C). Collectively, these results showed that PPARα is essential for the expression of *VNN1* in chicken liver cells.

### MiRNAs targeting the 3′UTR of the *VNN1* gene

**Figure 5:**
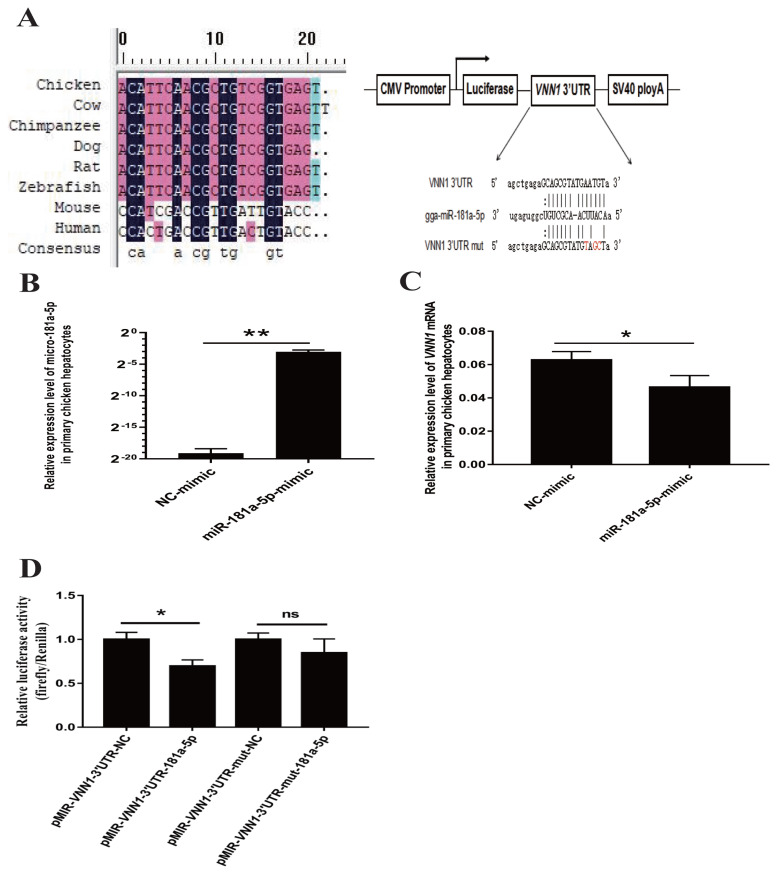
MiR-181a-5p downregulates vanin1 (*VNN1*) gene expression via targeting *VNN1*-3′UTR. (A) Mature microRNA-181a sequences of various species were searched using CoGeMiR (http://cogemir.tigem.it/), and DNAMAN software was used to analyze the conservation of microRNA-181a among multiple species. The top panel shows the structure and cloning sites of the pMIR-reporter vector. The wild-type and miR-181a-5p-binding site-mutated *VNN1*-3′UTR were cloned into the reporter vector. The bottom panel shows the complementarity between miR-181a-5p and the predicted target site in the *VNN1*-3′UTR. (B) and (C) Two-week-old primary hepatocytes were transfected with giga-miR-181a-5p mimics and negative controls (NC-mimic). After 48 h of transfection, RNA was extracted, and the expression of gga-miR-181a-5p and *VNN1* mRNA was detected by real-time PCR. ** p<0.01, n = 3. (D) gga-miR-181a-5p and NC-mimic were co-transfected into CHO-k1 cells with a luciferase reporter vector containing the corresponding target site or mutant target site, and relative luciferase activity was measured after 48 h of transfection. pRL-CMV (Renilla luciferase) was used as an internal control. Relative luciferase activity was determined as firefly luciferase activity normalized to Renilla luciferase activity. * p<0.05. PCR, polymerase chain reaction.

To clarify the posttranscriptional regulation mechanism of *VNN1*, bioinformatics analysis of the 3′UTR sequence was performed to identify putative miRNAs that target the 3′UTR of the *VNN1* gene. Based on the criteria of a score above 160 and free energy below −15 kcal/Mol, miR-181a-5p targeting the 3′UTR of *VNN1* mRNA was screened with miRanda v3.3 software for validation analysis. The 3′UTR of *VNN1* was predicted to interact stably with miR-181a-5p because it contains sequences that are completely complementary to the first 7 nucleotides of miR-181a-5p (Figure 5A). In addition, the mature miR-181a-5p sequence is highly conserved in various species, including cow, chimpanzee, dog, rat, and zebrafish. However, it is not conserved in humans and mice (Figure 5A).

### Overexpression of miR-181a-5p downregulates *VNN1* gene expression in primary chicken hepatocytes

To detect whether the expression of the *VNN1* gene was regulated by miR-181a-5p in the chicken liver, primary chicken hepatocytes were transfected with either the NC- or miR-181a-5p-mimic, and the expression of miR-181a-5p and *VNN1* mRNA was then further analyzed by real-time qRT-PCR. The results showed that transient transfection of the miR-181a-5p-mimic significantly increased the expression of miR-181a-5p in primary chicken hepatocytes (p< 0.01) (Figure 5B). As a result, the overexpression of miR-181a-5p significantly decreased the expression of *VNN1* mRNA (p<0.05) (Figure 5C). This result indicated the possibility that miR-181a-5p negatively regulates the expression of *VNN1* in chicken hepatocytes.

### Verification of the interaction between miR-181a-5p and *VNN1*

To verify whether miR-181a-5p directly targets the *VNN1* transcript, the wild-type reporter vector pMIR-*VNN1*-3′UTR and the mutant reporter vector pMIR-*VNN1*-3′UTR-mut were constructed as described in the Materials and Methods. Notably, the three bases in the pMIR-*VNN1*-3′UTR-mut plasmid identified in response to the predicted miR-181a-5p seed region were mutated by the overlap-PCR method. The pMIR-*VNN1*-3′UTR or pMIR-*VNN1*-3′UTR-mut was transfected into CHO-K1 cells together with the miR-181a-5p-mimic or the NC mimic. The miR-181a-5p-mimic significantly decreased the luciferase activity of *VNN1*-3′UTR, but it did not affect the activity of *VNN1*-3′UTR-mut, which demonstrated that miR-181a-5p directly bound to the predicted target site of the *VNN1* 3′UTR to affect luciferase expression (Figure 5D).

## DISCUSSION

Abdominal fat deposition is one of the most prominent issues in livestock production. It can cause a series of physiological disorders such as obesity, ascites and overall immunity declines and can lead to an increase in the cost of farming, which reduces the level of profitable agriculture development [21]. To a certain extent, chicken liver lipid metabolism and abdominal fat deposition are regulated by genetic factors [22]. Therefore, identifying new genetic factors and studying their biological functions and regulatory mechanisms is a good strategy for reducing fat deposition in the poultry abdomen. For example, Park et al [23] have revealed that chicken G0/G1 switch gene 2 (G0S2) plays an important role in abdominal fat deposition in chicken through the CRISPR-Cas9 system.

VNN1 is a pantetheinase that catalyzes the hydrolysis of pantetheine to produce pantothenic acid (vitamin B_5_) and cysteamine (highly effective antioxidant) [1]. Initial studies suggested that Vanin1 may promote an inflammatory response. Martin et al [24] showed that Vanin-1^−/−^ mice exhibited improved control of NSAID- or *Schistosoma*-induced intestinal inflammation and intestinal injury. Berruyer et al [3] further demonstrated that Vanin-1 licenses the production of inflammatory mediators by intestinal epithelial cells. Recent studies have revealed additional functions of *VNN1* gene. For example, van Diepen et al [5] revealed that RNAi-induced *VNN1* knockdown in mice aggravated the accumulation of liver TGs. Chen et al [7] characterized *VNN1* as a novel factor that activates hepatic gluconeogenesis. Our previous studies also showed that the *VNN1* gene is specifically expressed in chicken liver and negatively regulated by microRNA-122. These findings indicate that *VNN1* is likely to be a potential novel molecule involved in liver glycolipid metabolism. Thus, the molecular mechanisms that regulate *VNN1* in the chicken liver need to be clearly understood to evaluate its roles in liver glycolipid metabolism. In this study, we identified regulatory elements of the *VNN1* promoter and the *VNN1* 3′-UTR and validated the roles of PPARα and miRNA-181a-5P in the regulation of the chicken *VNN1* gene.

The chicken *VNN1* gene consists of 7 exons, which is consistent with the human and mouse *VNN1* genes. Studies in humans and mice have shown that the 1 kb and 3.5 kb promoter regions upstream of the *VNN1* gene exhibit strong transcriptional activity [8,12]. In this experiment, the ECR browser was used to predict the conservation of the chicken *VNN1* gene coding sequence and its upstream region. The results showed that the exons and ECRs of the chicken *VNN1* gene were highly conserved in relation to the human, mouse, rat and rhesus monkey sequences. The upstream sequence of the chicken *VNN1* gene did not show a conserved region shared with other species, but the 2.8 to 3.3 kb upstream region of the human, mouse and rhesus *VNN1* genes was highly conserved, indicating a large difference in the upstream regulatory region of the *VNN1* gene between poultry and mammals. To determine the TSS of the chicken *VNN1* gene, 5′-RACE was performed. Compared with the chicken *VNN1* genome sequence published in the NCBI database, we found that the *VNN1* TSS (chr3:56080813) was located 42 bp upstream of the translation initiation codon ATG (chr3:560 80855). To better understand the regulation of *VNN1* at the transcriptional level, we cloned and functionally characterized the 5′-regulatory region spanning the −1,000 to +200 nucleotides. The deletion analysis of the 5′-regulatory region showed that the P-548 fragment might be a core functional promoter, and the results were similar to those for the mouse *VNN1* core promoter region. Moreover, this is consistent with previous findings that the core promoter is usually located near the TSS [25].

In previous studies, comparative analysis of the proximal and distal promoters showed that several regions are conserved in mammals and that there are many predicted transcription factor-binding sites in the promoter region of the *VNN1* gene, including HNF4α, antioxidant response element, steroidogenic factor-1, sex-determining region Y box protein 9 and PPARg [7,8,26,27]. In the present study, we found potential transcription factor-binding sites for CEBPα and PPARα in the core promoter of the chicken *VNN1* gene located within the −421 to +127 sequence using bioinformatics analysis. PPARα is a member of the nuclear receptor superfamily and binds to the promoter regions of target genes to regulate many metabolic processes, particularly those involved in fatty acid oxidation. In the starvation experiment, the expression of the *VNN1* gene and the *PPARα* gene showed the same trend. Moreover, *VNN1* has been described as a putative PPARα target gene in the murine liver [28]. Therefore, we are particularly interested in whether PPARα affects the expression of the *VNN1* gene in chicken liver. Our results showed that GW7647, which was used as a high-potency, specific PPARα agonist, could promote the expression of the *VNN1* gene in a concentration-dependent manner. Moreover, siRNA-induced PPARα knockdown significantly reduced *VNN1* mRNA levels. Collectively, these findings showed that PPARα is essential for the expression of *VNN1* in chicken liver cells. To further elucidate the molecular mechanism by which PPARα regulates *VNN1* gene expression, a series of luciferase and site-directed mutagenesis experiments were performed. Our research revealed that PPARα increased the transcriptional activity of *VNN1* through binding to the putative PPARα-binding site located in the −49/−31 region of the *VNN1* gene promoter in two different types of cell lines. These results reveal that PPARα is a key transcription factor regulating *VNN1* expression in chicken hepatocytes. To our knowledge, this is the first report to demonstrate PPARα-mediated regulation of *VNN1* in chickens.

A large number of studies have confirmed that miRNAs can repress the expression of target mRNAs resulting in a posttranscriptional regulation pattern in animals. Our previous studies revealed that miR-122, as a target microRNA of the *VNN1* gene, can bind to the *VNN1* 3′UTR to reduce its expression in chicken hepatocytes. An interesting study showed that one miRNA can regulate the expression of multiple genes, and the expression of one gene can also be regulated simultaneously by multiple miRNAs [29]. In this paper, we provide experimental evidence suggesting that miR-181a-5p regulates the expression of the *VNN1* gene in chicken hepatocytes. MiR-181a-5p, a member of the miR-181 family, can regulate the expression of hundreds of genes, including cell proliferation-related genes (such as TGF-beta receptor type-1), apoptosis-related genes (such as myeloid cell leukemia-1), and cell differentiation-related genes (such as Nanog) [30,31]. Several recent reports indicate that miR-181a-5p is also widely involved in adipocyte differentiation and liver lipid metabolism. Three different groups revealed that miR-181a can regulate porcine preadipocyte and 3T3-L1 cell differentiation by directly targeting TGFBR1, tumor necrosis factor α (TNF-α), mothers against decapentaplegic homolog 7 and transcription factor 7 like 2 [32–34], thus affecting adipogenesis. In addition, Chu et al [35] revealed that miR-181a can regulate lipid metabolism via a novel miR-181a-IDH1 axis. In this study, overexpression of miR-181a-5p resulted in decreased expression of *VNN1* at the transcriptional level in primary chicken hepatocytes. Based on computer predictions, we found that miR-181a-5p can bind to the *VNN1* 3′UTR. Furthermore, dual-luciferase reporter assays and site mutation analyses verified that *VNN1* is a target gene of miR-181a-5p in chickens. As described above, the *VNN1* gene is widely involved in hepatic lipid metabolism in mammals. In poultry, CRISPR-Cas9- or RNAi-mediated knockdown of the *VNN1* gene in chicken hepatocytes significantly affects downstream lipid metabolism signals. Therefore, our findings may provide a new strategy for regulating lipid metabolism via the miR-181a-5p-*VNN1* axis.

In conclusion, our results strongly demonstrated that the predicted PPARα-binding site located in the −49/−31 region of the *VNN1* gene promoter is indeed functional and plays a crucial role in the pre-transcriptional regulation of the *VNN1* gene. Thus, we conclude that *VNN1* is a target gene of miR-181a-5p in chickens, and we propose that *VNN1* regulates lipid metabolism via the miR-181a-5p-*VNN1* axis.
