# Supplementary material for: Regulation of chicken vanin1 gene expression by peroxisome proliferators activated receptor α and miRNA-181a-5p
Source: Anim Biosci. 2020 Apr 12;34(2):172–84. doi: 10.5713/ajas.19.1000 (PMC7876720; doi:10.5713/ajas.19.1000)
Supplement: Supplementary file 1 [file ajas-19-1000-suppl.pdf]

Supplementary Figure 1.

DF-1 cells were transiently transfected with the P-263-luc and P-263-mut-luc constructs either alone or together with the PPARα expression plasmid (pcDNA3.1-PPARα) or pcDNA3.1. Luciferase activity was normalized to Renilla luciferase activity. The experiment was performed in triplicate wells and repeated in three independent trials. The data are presented as the means ± SEMs. ****, p<0.001 vs the P-263-mut-luc group,####, p<0.0001 vs the P-263-mut-luc group,$$$$,p<0.0001, pcDNA3.1-PPARα vs pcDNA3.1.

Supplementary Figure 2.

LMH cells (1.5×10^5 cells/well) were plated in 24-well plates for 24 h and grown to ~70% confluence in Waymouth's medium containing 10% fetal bovine serum. The medium was changed to Waymouth's medium containing 0.1% fetal bovine serum in the treatment group. After 24 h of serum starvation, total cellular RNA was extracted, and the expression of related genes was analyzed by RT-qPCR. The bars represent the means ± SEMs from three independent experiments. **, p < 0.01. β-Actin was used as a reference for normalization.

Supplementary Figure 3.

The isolated chicken hepatocytes were treated with different concentrations of GW7647 (0.5 μM, 1 μM, 2 μM) for 24 h. Total RNA was extracted, and the *ACOX1* gene was detected by RT-qPCR. The bars represent the means ± SEMs from three independent experiments. *, p < 0.05 and **, p < 0.01 vs the DMSO group (first bar). β-Actin was used as a reference for normalization.

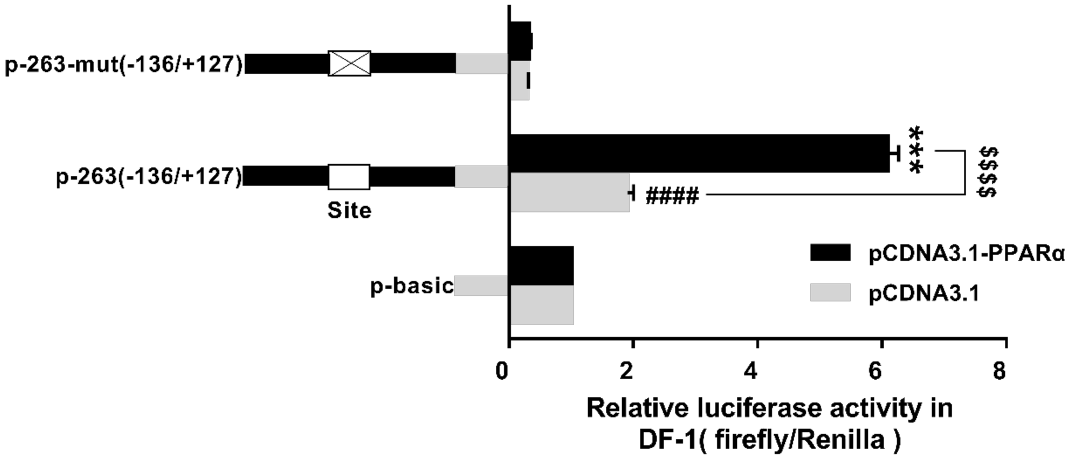

Supplementary Figure 1. PPARα upregulates *VNN1* promoter activity in DF-1 cells

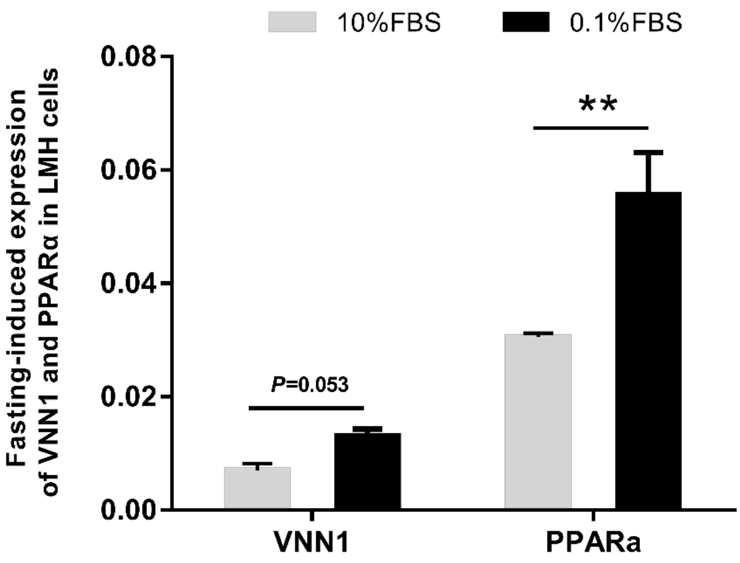

Supplementary Figure 2. This correlation between PPARα and VNN1 gene expression in serum-starved LMH cells

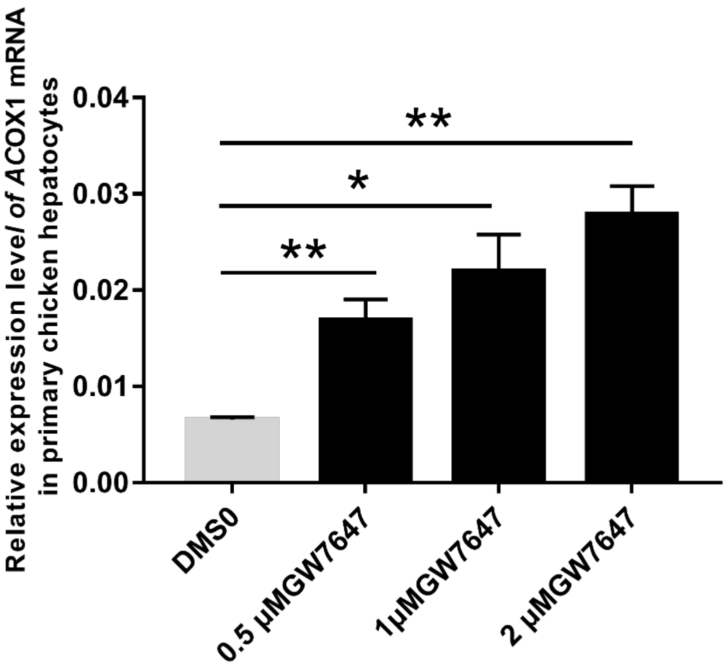

Supplementary Figure 3. GW7647 increased the ACOX1 mRNA expression level r in chicken primary hepatocytes

Supplementary Table S1 Primers used in this study

Primer name	Primer Sequence (5′–3′)	Primer purpose
5' RACE Outer Primer	CGCGGATCCACAGCCTACTGATGATCAGTCGATG	5' RACE
5' RACE Inner Primer	CATGGCTACATGCTGACAGCCTA	5' RACE
gga-VNN1-spR1	CTGTTCATCAGGGCCAAAGC	5' RACE
gga-VNN1-spR2	AAGCATCAGCAGGAGAAACC	5' RACE
gga-VNN1-F1	GG**GGTACC**TTACTGCAGAAACTCCATCC	Plasmid construction
gga-VNN1-F2	GG**GGTACC**GCGTTTCTGTCTTTCCTGAG	Plasmid construction
gga-VNN1-F3	GG**GGTACC**GAATGTTGTTGGAGGTAGGG	Plasmid construction
gga-VNN1-F5	GG**GGTACC**CCCTTTTCACCATTTCTCCG	Plasmid construction
gga-VNN1-R	CCC**AAGCTT**GCTGCGATGAAGGTGTCTGA	Plasmid construction
PPARα-Mut-Forward	AGTTGAACCTGCTCCGACTTATTTTC	Site mutation
PPARα-Mut-Reverse	GAAAATAAGTCGGAGCAGGTTCAACT	Site mutation
gga-VNN1-3'UTR-F	CC**GAGCTC**ATGCTGATGAGTGGGAGG	Plasmid construction
gga-VNN1-3'UTR-R	CCC**AAGCTT**TGATGCCAACAACTGAAA	Plasmid construction
VNN1-3'UTR-181-5p-mut-F	GAGAGCAGCGTATGTAGCTAATTTGAATTTTG	Site mutation
VNN1-3'UTR-181-5p-mut-R	CAAAATTCAAATTAGCTACATACGCTGCTCTC	Site mutation
gga -VNN1-qF	GACTCTGAAGGGAAACTGGT	RT-qPCR
gga -VNN1-qR	CAAAGCAGGTGAAAACGCCA	RT-qPCR
gga-β-actin-qF	CACGGTATTGTCACCAACTG	RT-qPCR
gga-β-actin-qR	ACAGCCTGGATGGCTACATA	RT-qPCR
gga-PPARα-qF	AGGAGAACCATCCGATTGA	RT-qPCR
gga-PPARα-qR	CTCAGACCTTGGCATTCGT	RT-qPCR
gga-ACOX1-qF	TTAATGACCCTGACTTCCAGC	RT-qPCR
gga-ACOX1-qR	CGATGAACAAAGCTTTTAAACCAG	RT-qPCR

Note: The mutation sites were indicated in underline. The nucleotides in bold font are restriction sites.
